# TEVAR for ruptured descending thoracic aortic aneurysm: case report

**DOI:** 10.1590/1677-5449.006716

**Published:** 2016

**Authors:** Sthefano Atique Gabriel, Enrico Rinaldi, Marco Leopardi, Germano Melissano, Roberto Chiesa

**Affiliations:** 1 Vita-Salute University School of Medicine, San Raffaele Scientific Institute, Fellow of Advanced Aortic Surgery, Milan, Italy.; 2 Vita-Salute University School of Medicine, San Raffaele Scientific Institute, Vascular Surgery, Milan, Italy.

**Keywords:** thoracic aortic aneurysm, ruptured aneurysm, endovascular procedures, aneurisma da aorta torácica, aneurisma roto, procedimentos endovasculares

## Abstract

A ruptured descending thoracic aortic aneurysm (rDTAA) is a life-threatening condition associated with high morbidity and mortality. Endovascular treatment for rDTAA promotes effective aneurysm exclusion with a minimally invasive approach. The authors report a case of a 76-year-old man with hemodynamically unstable 9-cm-diameter rDTAA treated with emergency thoracic endovascular aortic repair (TEVAR).

## Resumo

O aneurisma roto de aorta torácica descendente constitui uma situação ameaçadora associada a alta morbidade e mortalidade. O tratamento endovascular desse tipo de aneurisma promove exclusão eficaz com uma terapêutica minimamente invasiva. Os autores relatam o caso de um paciente do sexo masculino, 76 anos, hemodinamicamente instável, com aneurisma roto de aorta torácica descendente de 9 cm de diâmetro, tratado em caráter emergencial por cirurgia endovascular.

## INTRODUCTION

Ruptured descending thoracic aortic aneurysm (rDTAA) accounts for approximately 30% of all thoracic aortic ruptures and is a life-threatening condition associated with very high rates of morbidity and mortality (up to 97%).^1^^,^ 2 Very few patients are usually admitted to emergency care units alive and a small percentage of these patients survive the operation.^1^^-^ 3

The traditional treatment is aneurysm resection and replacement with an interposition of a prosthetic graft, as first described by Lam & Aram in 1951, but it remains a surgical challenge.^4^ Endovascular management of rDTAA, as first reported by Semba et al. in 1997, is less invasive than open repair and enables aneurysm exclusion without thoracotomy and aortic clamping.^5^ However, this approach is subject to anatomic and logistic limitations including constraints related to the quality of the landing zones, difficult iliac access and the need for a wide range of stent graft sizes to be available off-the-shelf for emergency use.^6^

The purpose of the present study is to report a case of thoracic endovascular aortic repair (TEVAR) for rDTAA and discuss some important issues related to this approach.

## CASE DESCRIPTION

A 76-year-old man was referred to our unit from a different hospital five hours after onset of acute and severe chest and back pain. On arrival the patient was awake, responsive, and hemodynamically unstable (arterial pressure: 80/50 mmHg). Laboratory test results showed hematocrit: 32.5%, hemoglobin: 10.6 g/dL, and creatinine: 1.29 mg/dL. The patient was hypertensive, a heavy smoker, and had no known history of aortic aneurysm or coronary artery disease.

**Figure 1:**
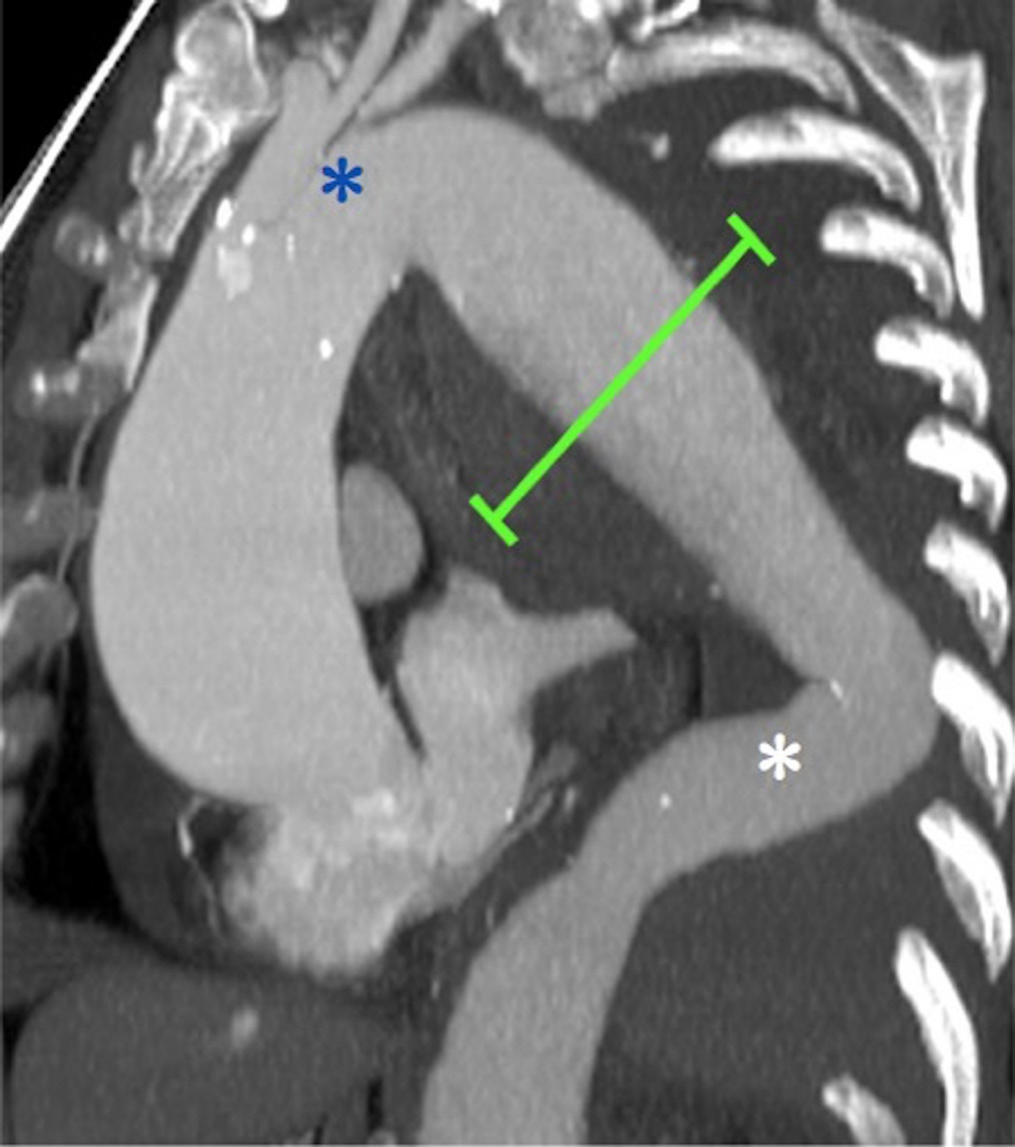
Preoperative CT MultiPlanar Reconstruction showing a 9-cm (line) ruptured descending thoracic aortic aneurysm with short and angulated proximal landing zone (upper asterisk) and an unclear and angulated distal landing zone (lower asterisk).

**Figure 2:**
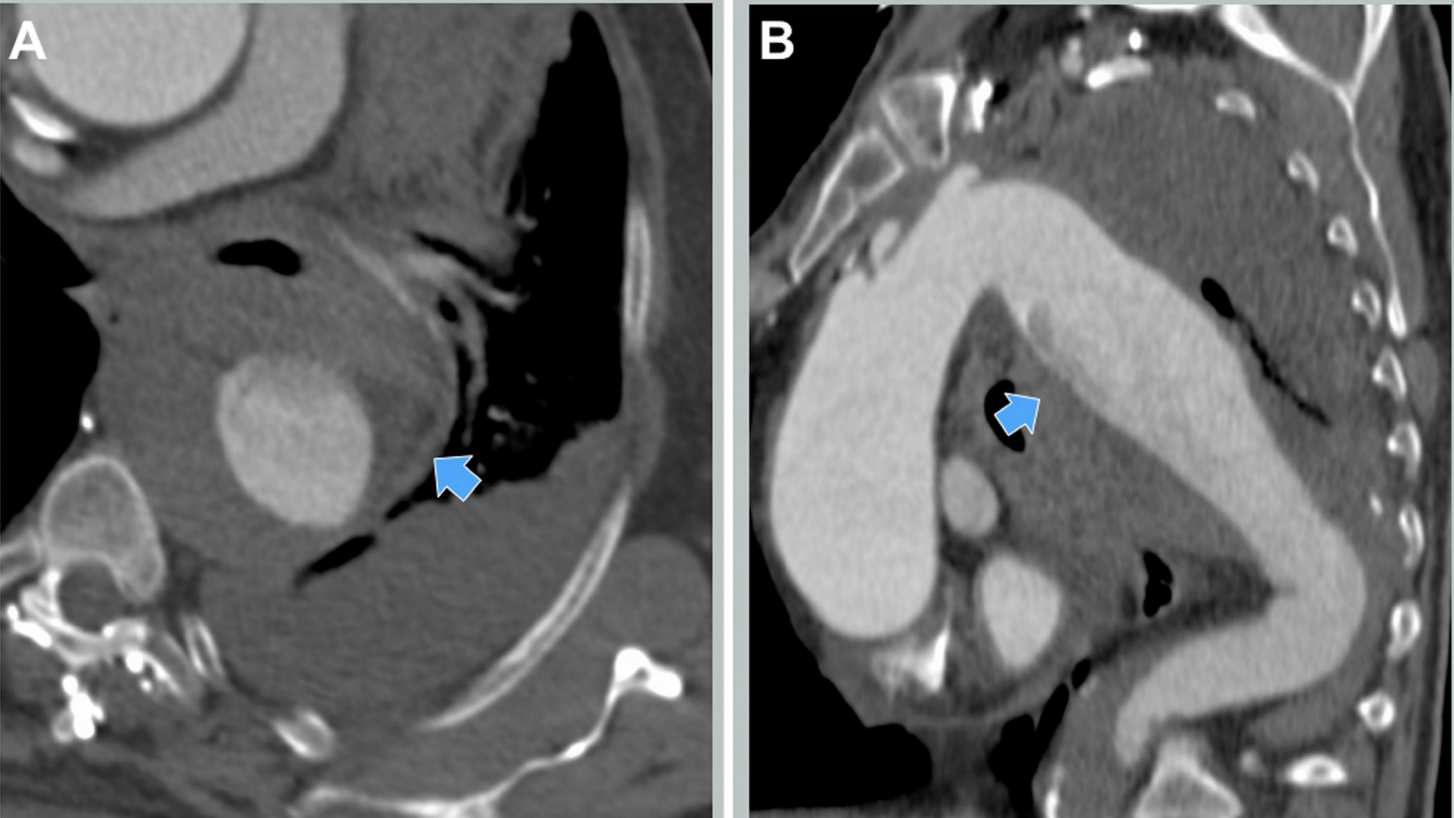
Preoperative CT-scan. (A) Axial view with periaortic hematoma (arrow); (B) Sagittal view with the possible point of descending thoracic aortic rupture indicated (arrow).

Transthoracic echocardiography demonstrated aortic insufficiency and pericardial effusion. Computed tomography (CT) performed at the referring center had shown a 9-cm-diameter rDTAA with periaortic hematoma and large amounts of blood in the intra-pleural and mediastinal cavities (Figures 1 and 2). Minutes after arrival, the patient went into frank shock (arterial pressure: 50/30 mmHg) and was subjected to an emergency TEVAR, performed immediately.

**Figure 3:**
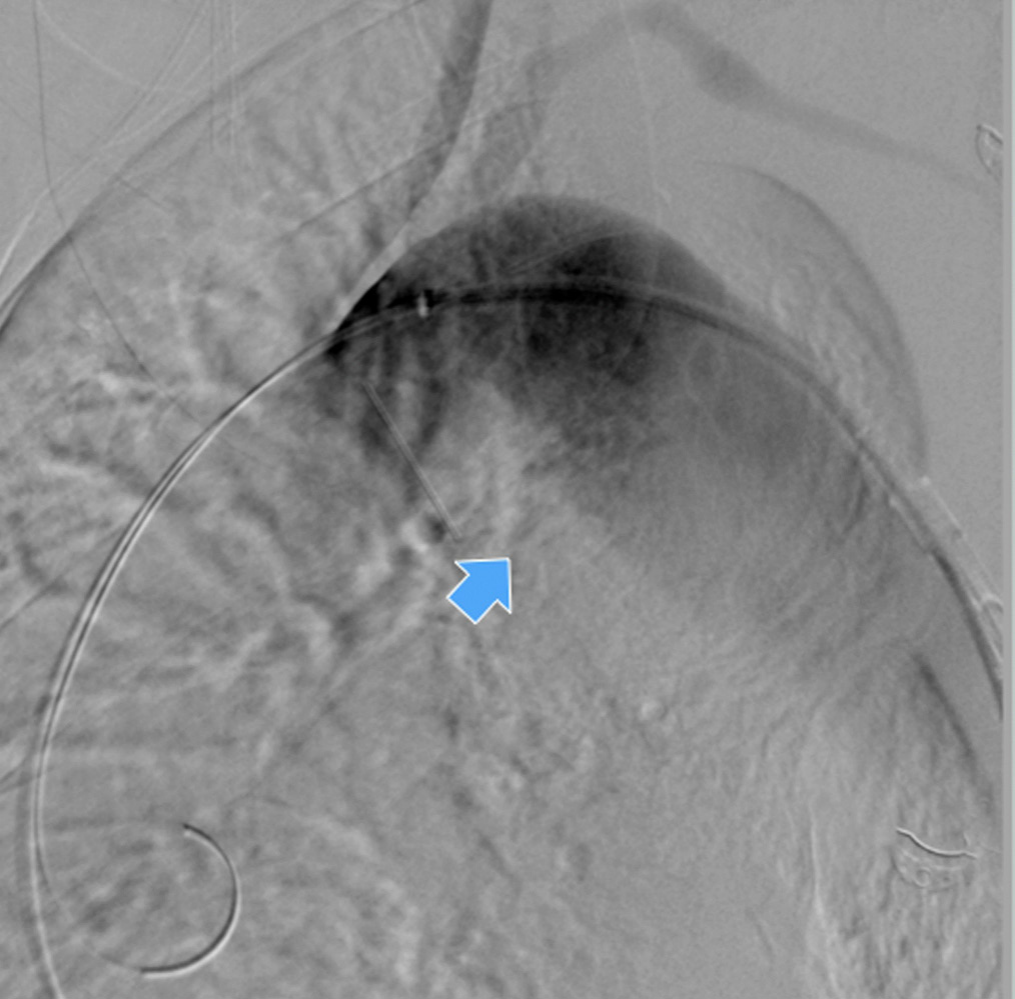
Intraoperative aortography with evidence of the descending thoracic aortic aneurysm (arrow).

The procedure was done under general anesthesia. Access to the left femoral artery was obtained percutaneously and the right femoral artery was surgically exposed with insertion of 8 Fr sheaths bilaterally. No systemic heparin was administered. An extra stiff Lunderquist guidewire was advanced through the right external iliac artery to the ascending aorta. An intraoperative aortography was obtained (Figure 3). A proximal Zenith Alpha™ Thoracic Endovascular Graft (42 _×_ 225 mm; Cook® Medical) was then advanced and deployed just distal to the left common carotid artery, with intentional occlusion of the left subclavian artery.

A second device, a distal Zenith Alpha™ Thoracic Endovascular Graft (46 _×_ 211 mm; Cook® Medical), was inserted, overlapping the first one. A completion angiography demonstrated both endoprostheses correctly positioned, total exclusion of the descending thoracic aortic aneurysm, no endoleaks, and a patent left common carotid. Transesophageal echocardiography revealed aneurysm exclusion and immediate aneurysm sac thrombosis. The entire procedure took 60 minutes and blood loss was trivial. The patient received 3 units of intraoperative blood transfusion.

**Figure 4:**
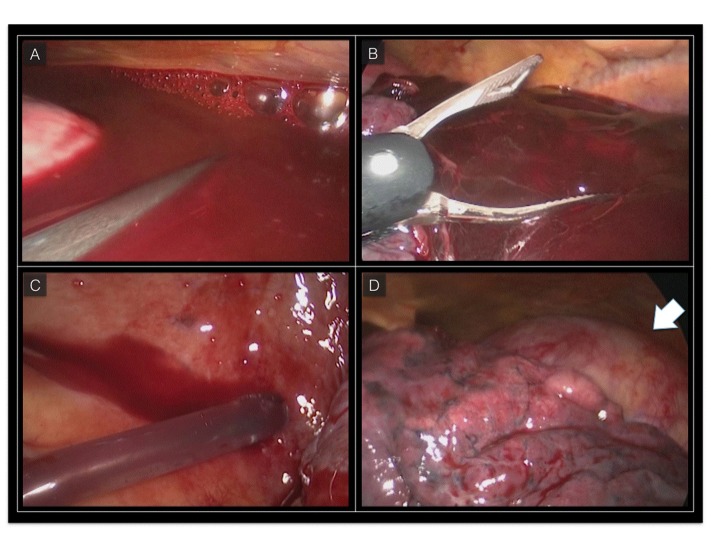
Videothoracoscopy. (A) Blood clot in the left pleural cavity; (B) Mechanical removal of the blood clot; (C) Complete aspiration of the blood in the left pleural cavity; (D) Left lung mobilization with thoracic aorta exposure (arrow), the thoracic aorta is completely excluded without any sign of residual bleeding.

**Figure 5:**
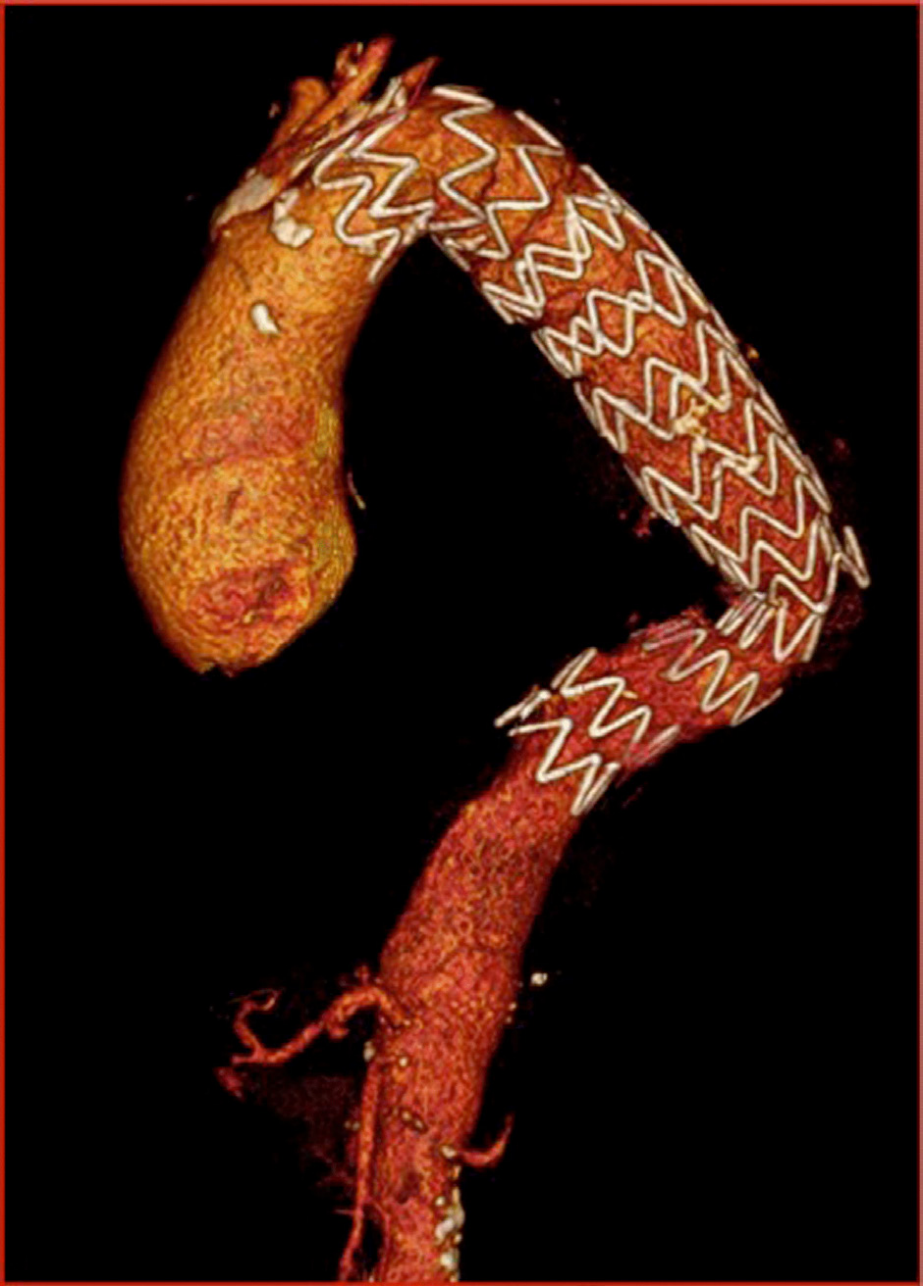
Postoperative CT scan showing correctly positioned endograft.

The patient was immediately awakened and was put on noninvasive ventilation. No neurologic complications were observed. Forty-eight hours after TEVAR, the patient underwent videothoracoscopy and approximately 1000 mL of blood clots were removed from the mediastinal cavity (Figure 4). The patient’s post-procedure course was clinically uneventful. A CT scan confirmed correct positioning of the stent-grafts and exclusion of the aneurysm with no endoleaks (Figure 5). The patient was discharged from the hospital on the 7th day after TEVAR. Three months later the patient underwent a successful aortic valve replacement. Four months after the procedure he is alive and well.

## DISCUSSION

Although open surgical repair remains the traditional treatment of rDTAA, TEVAR is a minimally invasive approach that does not requires thoracotomy, aortic cross-clamping, systemic heparin, cardiopulmonary bypass, or hypothermic cardiac arrest.^7^^,^ 8 Moreover, TEVAR enables safe and effective aneurysm exclusion, can be used for treatment of patients in critical conditions, and is associated with shorter operating times, minimal additional blood loss, and lower fluid requirements.^7^^,^ 8

In our patient, the CT scan was of sub-optimal quality and did not show the precise point of thoracic aortic rupture with active bleeding, however, the combination of hemomediastinum, hemothorax, hypovolemic shock, and thoracic aortic aneurysm suggested the diagnosis of rDTAA. Our option for TEVAR was based on patient’s hemodynamic status and the need for immediate intervention. The angulated aortic arch, the short and angulated proximal landing zone and an unclear distal landing zone were challenging technical features that we had to manage to perform a successful TEVAR.

Insertion of a modular endograft with separate proximal and distal components allowed us a longer coverage of the descending thoracic aorta with secure aneurysm exclusion, despite inadequate aortic endograft planning, and offered the possibility of focusing on the proximal and distal releases at two different times, with more precise deployment. Moreover, the overlapping junctions between graft components provided greater stability and good apposition of both endoprostheses in the aortic wall, despite the adverse anatomical characteristics such as mismatch between the proximal and distal sealing zones.

One feared complication after TEVAR for rDTAA is postoperative paraplegia. Jonker et al. observed permanent postoperative paraplegia in 2.2% of patients submitted to TEVAR for rDTAA, compared with 8.7% after open repair.^9^ Hogendoorn et al. explain that paraplegia after TEVAR is associated with non-reimplantation of intercostal arteries, graft coverage of the left subclavian artery, and compromised flow to collateral blood supply of the spinal cord by embolization of debris material dislodged from the aortic wall during catheter manipulation.^10^ Additionally, Chiesa et al. have reported that in emergency procedures, blood loss and perioperative hypotension are associated with the occurrence of spinal cord ischemia after elective TEVAR.^11^

However, Bavaria et al. have listed absence of aortic cross-clamping, fewer periods of perioperative hypotension due to blood loss or hemodynamic shifts, and slow thrombosis of the aneurysm sac compared with acute occlusion of intercostal vessels during surgical repair as possible explanations for the reduced risk of paraplegia after TEVAR for nonruptured DTAA.^12^

Surgical aspects that might have prevented postoperative neurological symptoms in our patient were the permissive hypotension during endoprostheses positioning, the aggressive fluid resuscitation after stent-graft deployment, the reduced operating time, the minimal intraoperative blood loss, and the immediate awakening of the patient. In case of paraparesis or permanent paraplegia after TEVAR for rDTAA, the recommendations are to maintain mean aortic pressure between 90 and 110 mmHg after stent deployment and cerebrospinal fluid pressure and central venous pressure below 10 mmHg.^10^^,^ 13

According to the Society for Vascular Surgery Practice Guidelines, for patients who needs emergency TEVAR for life-threatening acute aortic syndromes with coverage of the left subclavian artery, its revascularization should be individualized and addressed expectantly on the basis of anatomy, urgency, and availability of surgical expertise.^14^ In our patient, we opted for simple occlusion of the left subclavian artery during TEVAR in order to create an adequate proximal landing zone for stent-graft positioning and to effect immediate sealing of the point of aortic rupture.

Recent studies highlight a reduction in mortality for patients treated with TEVAR for rDTAA compared to open surgical repair. Jonker et al. reported a significantly lower 30-day mortality in patients treated with TEVAR for rDTAA compared to open repair (18.9 vs. 33.3%; p = 0.016, respectively).^15^ One year later, the same author observed that postoperative pulmonary complications (31.9 vs. 17.4%; p = 0.032, respectively), acute renal failure (24.6 vs. 8.7%; p = 0.006, respectively) and median length of hospital stay for surviving patients (22 days vs. 8 days; p < 0.001, respectively) were all significantly higher in patients treated with open surgery for rDTAA compared to TEVAR patients.^9^

Hemothorax is frequently associated with rDTAA and it may provoke compression of the esophagus and cardiovascular structures and compromises postoperative survival.^16^^,^ 17 Previous studies have reported that blood clots may adhere to the lung and pleura, making them difficult to remove with a single chest tube, and leading to respiratory insufficiency and thoracic infection.^18^^,^ 19 In our patient, hemothorax evacuation with chest drainage tube or thoracotomy would not have been possible due to the presence of organized blood clots. Therefore we opted for videothoracoscopy of the mediastinal cavity to remove the thoracic blood clots that had been retained.

Piffaretti et al. analyzed fifty-six patients with ruptures of the descending aorta and hemothorax who had been treated with TEVAR (traumatic rupture in 41%, atherosclerotic aneurysm in 36%, Debakey type III a dissection in 14% and penetrating aortic ulcer in 9%), concluding that prompt hemothorax evacuation reduced postoperative mortality in drained patients who had presented significantly worse pre-operative respiratory parameters.^20^

Possible limitations to TEVAR for rDTAA include: no proximal or distal aortic neck, an aortic diameter too wide for commercially available thoracic endografts, and severe aortic calcification and tortuosity.^9^^,^ 15 ^,^ 21 Postoperative endoleaks are responsible for a high reintervention rate (45.4%) after TEVAR for rDTAA and should be prevented with careful anatomical analysis of the thoracic aorta, meticulous preoperative stent-graft planning and precise endograft deployment.^9^^,^ 15 ^,^ 21 In our patient, transesophageal echocardiography showed that the aneurysm sac had undergone thrombosis immediately after TEVAR, with no endoleaks.

In conclusion, TEVAR is feasible for rDTAA. It can be performed in high-risk patients with adverse anatomical characteristics and represents a good option even in cases of sub-optimal diagnosis of thoracic aortic rupture. Hemothorax secondary to rDTAA should be drained.
